# A Case of Measles Virus-caused Subacute Sclerosing Panencephalitis Diagnosed by Molecular and Clinical Analysis

**DOI:** 10.1093/ofid/ofaf453

**Published:** 2025-08-28

**Authors:** Tingyan Liu, Caiyan Zhang, Meixiu Ming, Lian Chen, Weiming Chen, Yang Xu, Meili Shen, Jia Liu, Qiuxiang Ou, Hua Bao, Xiangjun Chen, Guoping Lu, Gangfeng Yan

**Affiliations:** Paediatric Intensive Care Unit, Children's Hospital of Fudan University, National Children's Medical Center, Shanghai, China; Paediatric Intensive Care Unit, Children's Hospital of Fudan University, National Children's Medical Center, Shanghai, China; Paediatric Intensive Care Unit, Children's Hospital of Fudan University, National Children's Medical Center, Shanghai, China; Department of Pathology, Children's Hospital of Fudan University, National Children's Medical Center, Shanghai, China; Paediatric Intensive Care Unit, Children's Hospital of Fudan University, National Children's Medical Center, Shanghai, China; Medical Department, Nanjing Dinfectome Technology Inc., Nanjing, Jiangsu, China; Medical Department, Nanjing Dinfectome Technology Inc., Nanjing, Jiangsu, China; Medical Department, Nanjing Dinfectome Technology Inc., Nanjing, Jiangsu, China; Medical Department, Nanjing Dinfectome Technology Inc., Nanjing, Jiangsu, China; Medical Department, Nanjing Dinfectome Technology Inc., Nanjing, Jiangsu, China; Department of Neurology, Huashan Hospital, Fudan University and Institute of Neurology, Fudan University, National Center for Neurological Disorders, Shanghai, China; Paediatric Intensive Care Unit, Children's Hospital of Fudan University, National Children's Medical Center, Shanghai, China; Paediatric Intensive Care Unit, Children's Hospital of Fudan University, National Children's Medical Center, Shanghai, China; School of Public Health and Shanghai Institute of Infectious Disease and Biosecurity, Fudan University, Shanghai, China

**Keywords:** electroencephalogram, magnetic resonance imaging, measles, metagenomic next-generation sequencing, SSPE

## Abstract

Subacute sclerosing panencephalitis (SSPE) is a rare and lethal neurodegenerative encephalitis caused by persistent measles virus infection. We report an SSPE case in a patient infected at 6 months old, diagnosed using clinical methods and metagenomic sequencing, highlighting the benefits of combining clinical and molecular techniques for improved diagnosis.

Subacute sclerosing panencephalitis (SSPE) is a life-threatening neurodegenerative encephalitis characterized by persistent measles virus (MeV) infection within the brain [1]. SSPE is an extremely rare disease, and its incidence is closely related to measles vaccination coverage in various regions, spanning from 0.06 per million in North America to 29 per million in Papua New Guinea [2]. About 4–18 cases of SSPE are expected per 100 000 MeV infections [3]. The typical manifestations of SSPE are seizures, behavioral changes, intellectual disabilities, paroxysmal movements, myoclonic jerks, and motor deficits [4]. Despite the widespread administration of the measles vaccine, SSPE continues to manifest sporadically among unvaccinated or immunocompromised individuals, particularly in children and young adolescents [5]. Currently, no validated treatment is available for SSPE, and patients mainly receive supportive therapies. Dyken's modiﬁed criteria have been used for SSPE diagnosis, which relies on the clinical history, the presence of measles antibody in the cerebrospinal fluid (CSF), the characteristic electroencephalogram (EEG) pattern, or histological findings in the brain biopsy [6]. Nevertheless, many of these tests have unsatisfying speciﬁcity and/or sensitivity to diagnose SSPE. Additionally, early SSPE symptoms are mainly nonspecific and have a delayed onset (ie, 4–10 years after the initial MeV infection), resulting in largely undiagnosed or misdiagnosed diseases [7]. In recent years, metagenomic next-generation sequencing (mNGS) technology has demonstrated promising advantages in the detection of rare or even unknown pathogen infections, which is difficult to accomplish by traditional approaches. Here, we present a compelling case of MeV-induced SSPE, wherein the etiological diagnosis was facilitated by mNGS technology.

On December 2021, a 6-year-old boy was transferred to our hospital presenting with abnormal behavior and jerk movements persisting for 3 months. The child initially exhibited speech regression characterized by decreased language output, slowed speech, and inability to understand words, and he later on progressed to additional symptoms including impaired sitting and walking, salivation, facial muscle twitches, excessive blinking, and incontinence. Considering the possibility of autoimmune encephalitis, the child had received one 10 g dose of immunoglobulin and 3 high doses of methylprednisolone between November 2021 and December 2021, prior to admission to our hospital (Supplementary Fig. 1). However, the child's condition was not significantly improved.

During the initial hospitalization period (from November 2021 to December 2021), the patient exhibited exceptionally elevated immunoglobulin G (IgG) antibody levels (2.51 g/L) in CSF, although the first mNGS analysis did not detect any pathogens in CSF. Before admission to our hospital, consecutive magnetic resonance imaging (MRI) scans revealed mild cerebral atrophy. The EEG displayed trace alternant during sleep (Figure 1*A*), which is unusual in infants but not in children. Given the patient's history of MeV infection at 6 months old without vaccination for measles, CSF samples were re-collected for anti-MeV IgG and the result turned out positive. Pending results, the child received 3 rituximabs infusions. However, due to rapid progression to convulsions and coma, the patient underwent plasma exchange and was transferred to the pediatric intensive care unit on December 2021.

**Figure 1.**
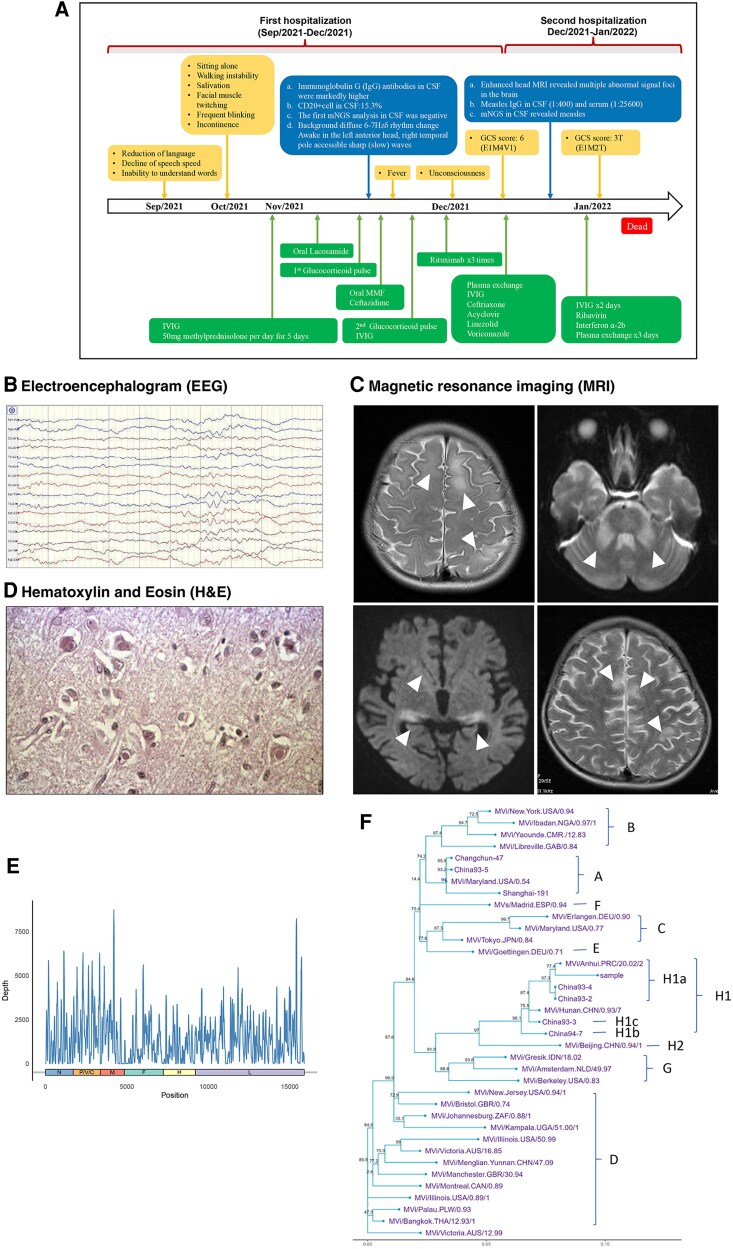
Various clinical/molecular diagnoses of the SSPE patient. *A*, The detailed diagnosis and treatment process of the patient. CSF, cerebrospinal fluid; IVIG, intravenous immunoglobulin; GCS, Glasgow Coma Scale; mNGS, metagenomic next-generation sequencing. *B*, The electroencephalogram (EEG) displayed trace alternant during sleep. *C*, Enhanced magnetic resonance imaging (MRI) revealed multiple abnormal signal foci (white arrows) in the brain. *D*, The Hematoxylin and Eosin (H&E) stained brain sections showed neuronophagia in brain tissue. *E*, The sequencing depth of mNGS from the brain tissue sample. *F*, The phylogenetic results showing that the brain tissue mNGS sample (labeled “sample” in the figure) formed a cluster with the H1a strain.

Subsequent enhanced head MRI after admission to our hospital revealed multiple abnormal signal foci in the brain (Figure 1*B*), and enzyme-linked immunosorbent assay (ELISA) showed a high titer of anti-MeV IgG antibodies in both CSF (1:400) and serum (1:25 600). Additionally, the mNGS identified MeV infection in CSF (MeV sequence reads: 11; MeV genome coverage: 2.2%). Based on this clinical evidence, the patient was diagnosed with SSPE, an exceedingly rare disease with persistent MeV infection.

The child received acyclovir and immunoglobulin therapy combined with intravenous immunoglobulin (IVIG) and plasma exchange after hospitalization, and then switched to ribavirin and interferon ɑ-2b since December 2021. However, no improvement was observed. Given the deteriorating conditions, the child's guardians opted to discontinue treatment, and the child was discharged, succumbing to the illness on January 2022. With consent from the guardians, autopsy samples from focal brain regions were subjected to further analysis. Histopathological examination revealed neuronophagia in brain tissue (Figure 1*C*), which is a gold standard for measles. Notably, mNGS analysis of brain tissues detected a high MeV load (MeV sequence reads: 226 618; MeV genome coverage: 97.45%). This result was further confirmed using Sanger sequencing. Considering the high coverage of tissue mNGS results, we conducted subsequent phylogenetic analysis and identified the closest related wild-type (WT) MeV strain as H1a. Notably, a total of 29 missense mutations in MeV were detected, including 2 (6.9%) from nucleocapsid (N) gene, 13 (44.8%) from phospho-protein (P) gene, 2 (6.9%) from matrix (M) gene, 1 (3.4%) from fusion (F) gene, 7 (24.1%) from hemagglutinin (H) gene, and 4 (13.8%) from large (L) gene (Supplementary Table 1).

Previous studies indicated that SSPE typically arose from MeV infections contracted earlier in life, remaining dormant for 4 to 10 years before developing into the disease state [3]. Our case aligned with this pattern, as the patient contracted MeV at 6 months old, exhibiting SSPE symptoms ∼5.5 years later. The hallmark manifestations of SSPE include a subacute decline in cognitive and behavioral conditions, followed by myoclonus, ataxia, seizures, and/or visual impairment [8]. Similarly, our patient initially presented with generalized tonic colonic convulsions, gait abnormality, and abnormal behavior with an incoordinate speech that gradually progressed to aphasia. Previous reports suggested that SSPE can progress rapidly after developing the initial symptoms, resulting in vegetative states or death within a short period of time [9].

The most effective precaution of SSPE is measles vaccination. In China, the routine measles immunization schedule includes 2 doses: the first at 8 months and the second at 18–24 months. These recommendations vary globally based on regional risk and maternal antibody prevalence. Unfortunately, our patient contracted MeV before the recommended vaccination age due to a local measles outbreak. Given the elevated SSPE risk in infants and the potential for maternal antibodies to interfere with vaccine-induced immunity when administered too early, the balance between early immunization and optimal immunogenicity must be carefully considered, and in high-risk settings, early vaccination may still be beneficial [10, 11]. Moreover, the recent surge in MeV infections, potentially exacerbated by the COVID-19 pandemic and rising vaccine refusal trend in the population [12], underscores the critical need to advocate vaccination for measles prevention and SSPE mitigation.

While timely treatment may benefit many SSPE patients, diagnostic challenges persist due to low awareness and nonspecific clinical presentations. Moreover, early SSPE often yields normal MRI findings [12], which was also observed in our patients. Dyken's modiﬁed criteria include 2 major markers (ie, clinical history and elevated CSF anti-MeV antibody) and 4 minor markers (ie, typical EEG, increased CSF IgG, brain biopsy, and molecular diagnostic tests), and 2 major and 1 minor criteria are required to diagnose SSPE [6]. The advent of mNGS presents a promising avenue for pathogen detection in CNS, offering sensitivity, high throughput, and comprehensive analysis [13]. Considering the medical history of our patient, subsequent CSF mNGS analysis, which was conducted with special care with no delay between sample collection and mNGS testing, detected 11 MeV reads (2.2% coverage) aiding the SSPE diagnosis of our patient. Notably, as with previous tissue-based diagnosis [14], we found brain tissue mNGS could provide superior sensitivity to detect MeV, resulting in 226 618 MeV reads and 97.45% coverage (the corresponding human reads were 56 870 999; the human/MeV read ratio was 0.00398). Another advantage of mNGS compared with traditional methods is that it can help determine the genotype and the specific pathogenic mutations of MeV, given that the MeV genome undergoes a series of mutations in CNS, particularly in M and F genes, that ultimately lead to SSPE [8]. Through tissue mNGS, our patient was determined to be infected with H1a MeV alongside missense mutations in M and F genes and 4 other MeV genes, offering insights into SSPE etiology and progression. Clusters of mutations identified around the matrix (M) protein in many SSPE viruses suppress productive infectious particle release and accelerate cell–cell fusion, which are features of SSPE viruses. In addition, neuropathogenic is closely related to the character of the viral fusion (F) protein, and amino acid substitution(s) in the F protein of some SSPE viruses confers F protein hyperechogenicity, facilitating viral propagation in the CNS through cell–cell fusion and leading to neurovirulence [15].

In conclusion, SSPE represents a rare yet devastating disease stemming from complications of childhood measles. As a neurodegenerative condition, SSPE can culminate in the patient's demise in the absence of timely intervention. Our case study underscores the significance of leveraging advanced technologies for CNS pathogen identification. Specifically, it highlights the potential clinical utility of mNGS to provide precise, prompt, and comprehensive SSPE diagnoses.

## Supplementary Material

ofaf453_Supplementary_Data

## Supplementary Data

Supplementary materials are available at *Open Forum Infectious Diseases* online. Consisting of data provided by the authors to benefit the reader, the posted materials are not copyedited and are the sole responsibility of the authors, so questions or comments should be addressed to the corresponding author.
