# Supplementary material for: A Case of Measles Virus-caused Subacute Sclerosing Panencephalitis Diagnosed by Molecular and Clinical Analysis
Source: Open Forum Infect Dis. 2025 Aug 28;12(8):ofaf453. doi: 10.1093/ofid/ofaf453 (PMC12393149; doi:10.1093/ofid/ofaf453)
Supplement: ofaf453_Supplementary_Data [file ofaf453_supplementary_data.docx]

**Supplementary Methods**

**Clinical monitoring and examination**

Clinical monitoring and examination were conducted according to clinical diagnosis and treatment needs. Computed tomography (CT) was performed using GE Lightspeed VCT scanner. MRI was performed using a 1.5T Siemens Magnetom Avanto scanner. EEG was recorded by Neurofax EEG-1200C. Autoimmune encephalitis entibodies were detected using double fluorescence method. CSF electrophoresis of IgG oligoclonal bands was also conducted. Clinical microbial tests were conducted to diagnosis pathogens, included culture of blood, CSF, sputum and urine, PCR of herpes simplex virus and enterovirus, serology tests of HSV, human gammaherpesvirus 4 (EBV), human betaherpesvirus 5 (CMV), Primate erythroparvovirus 1, rubella virus, Toxoplasma, and measles virus, 1,3-β-D glucan (G) test, tuberculosis infection T cell spot (T-spot), and Cryptococcal antigen (CrAg).

**mNGS**

CSF DNA was extracted using the TIANamp Magnetic DNA Kit (Tiangen, China), and brain tissues DNA was extracted using the QIAamp DNeasy Blood & Tissue Kit (QIAGEN, Germany). RNA was extracted using the QIAamp Viral RNA Mini Kit (QIAGEN, Germany) according to the manufacturer’s protocol. After quantitative and qualitative evaluation of DNA and RNA, a DNA library was constructed using Hieff NGS OnePot II DNA Library Prep Kit (Yeasen Biotech, China), and an RNA library was constructed by the VAHTS Universal V8 RNA-seq Library Prep Kit (Vazyme Biotech, China), according to the manufacturers’ protocol. The use of DNA and RNA dual processes for mNGS was to comprehensively detect possible pathogens (including both DNA and RNA pathogens). Finally, we completed sequencing in the single-end 50 bp sequencing mode using MGISEQ-200 (BGI, China). No template negative controls (NTCs) were included in the extraction, library preparation, and sequencing process. Raw sequencing data was split by bcl2fastq2, and high-quality sequencing data were generated using Trimmomatic by removing low-quality, adapter contaminated, duplicated, and short (<36 bp) reads. Human host sequences were subtracted by mapping to the human reference genome (hs37d5) using bowtie2. Reads that could not be assigned to the human genome were retained and aligned with the microorganism genome database for microbial identification using Kraken 2, and the species abundance were estimated by Bracken. The microorganism genome database containing genomes or scaffolds of bacteria, fungi, viruses, and parasites were downloaded from GenBank (release 238, <ftp://ftp.ncbi.nlm.nih.gov/genomes/genbank/>). The phylogenetic tree of MeV was built based on Wang H. *et al*. (1), and the data were obtained from Genetic diversity of wild-type measles viruses and the global measles nucleotide surveillance database (‎MeaNS). The reference sequencing data were obtained from NCBI (accession: NC_001498.1).

**Table S1. MeV missense mutations that were detected in the brain tissue using mNGS.**

| Accession # | Position | Reference | Altered | Depth | Effect | Gene | Feature ID | Nucleotide | Amino acid | Product |
| --- | --- | --- | --- | --- | --- | --- | --- | --- | --- | --- |
| NC_001498.1 | 50 | A | G | 68 | . | . | . | . | . |  |
| NC_001498.1 | 143 | A | G | 68 | synonymous | MeVgp1 | rna-MeVgp1 | c.36A>G | p.Lys12Lys | nucleocapsid protein |
| NC_001498.1 | 538 | C | T | 47 | missense | MeVgp1 | rna-MeVgp1 | c.431C>T | p.Ser144Phe | nucleocapsid protein |
| NC_001498.1 | 575 | A | G | 46 | synonymous | MeVgp1 | rna-MeVgp1 | c.468A>G | p.Glu156Glu | nucleocapsid protein |
| NC_001498.1 | 680 | G | A | 157 | synonymous | MeVgp1 | rna-MeVgp1 | c.573G>A | p.Ser191Ser | nucleocapsid protein |
| NC_001498.1 | 725 | T | C | 335 | synonymous | MeVgp1 | rna-MeVgp1 | c.618T>C | p.Val206Val | nucleocapsid protein |
| NC_001498.1 | 770 | C | T | 121 | synonymous | MeVgp1 | rna-MeVgp1 | c.663C>T | p.Asn221Asn | nucleocapsid protein |
| NC_001498.1 | 944 | T | C | 98 | synonymous | MeVgp1 | rna-MeVgp1 | c.837T>C | p.Thr279Thr | nucleocapsid protein |
| NC_001498.1 | 1085 | A | G | 39 | synonymous | MeVgp1 | rna-MeVgp1 | c.978A>G | p.Ala326Ala | nucleocapsid protein |
| NC_001498.1 | 1091 | A | G | 99 | synonymous | MeVgp1 | rna-MeVgp1 | c.984A>G | p.Ser328Ser | nucleocapsid protein |
| NC_001498.1 | 1268 | T | A | 69 | synonymous | MeVgp1 | rna-MeVgp1 | c.1161T>A | p.Thr387Thr | nucleocapsid protein |
| NC_001498.1 | 1324 | T | C | 49 | missense | MeVgp1 | rna-MeVgp1 | c.1217T>C | p.Ile406Thr | nucleocapsid protein |
| NC_001498.1 | 1328 | T | C | 11 | synonymous | MeVgp1 | rna-MeVgp1 | c.1221T>C | p.Ser407Ser | nucleocapsid protein |
| NC_001498.1 | 1616 | A | G | 49 | synonymous | MeVgp1 | rna-MeVgp1 | c.1509A>G | p.Gly503Gly | nucleocapsid protein |
| NC_001498.1 | 1620 | T | C | 17 | synonymous | MeVgp1 | rna-MeVgp1 | c.1513T>C | p.Leu505Leu | nucleocapsid protein |
| NC_001498.1 | 1717 | G | A | 82 | . | . | . | . | . |  |
| NC_001498.1 | 1879 | G | A | 57 | missense | MeVgp2 | rna-MeVgp2 | c.73G>A | p.Gly25Ser | V protein |
| NC_001498.1 | 1895 | A | G | 133 | missense | MeVgp2 | rna-MeVgp2 | c.89A>G | p.Glu30Gly | V protein |
| NC_001498.1 | 1938 | A | G | 219 | synonymous | MeVgp2 | rna-MeVgp2 | c.132A>G | p.Gly44Gly | V protein |
| NC_001498.1 | 1944 | C | G | 213 | missense | MeVgp2 | rna-MeVgp2 | c.138C>G | p.Asp46Glu | V protein |
| NC_001498.1 | 1988 | G | A | 138 | missense | MeVgp2 | rna-MeVgp2 | c.182G>A | p.Ser61Asn | V protein |
| NC_001498.1 | 1993 | C | T | 49 | missense | MeVgp2 | rna-MeVgp2 | c.187C>T | p.Pro63Ser | V protein |
| NC_001498.1 | 2017 | A | G | 100 | missense | MeVgp2 | rna-MeVgp2 | c.211A>G | p.Thr71Ala | V protein |
| NC_001498.1 | 2167 | A | G | 146 | missense | MeVgp2 | rna-MeVgp2 | c.361A>G | p.Lys121Glu | V protein |
| NC_001498.1 | 2201 | A | G | 154 | missense | MeVgp2 | rna-MeVgp2 | c.395A>G | p.Gln132Arg | V protein |
| NC_001498.1 | 2256 | C | T | 51 | synonymous | MeVgp2 | rna-MeVgp2 | c.450C>T | p.Asn150Asn | V protein |
| NC_001498.1 | 2403 | A | G | 87 | synonymous | MeVgp2 | rna-MeVgp2 | c.597A>G | p.Arg199Arg | V protein |
| NC_001498.1 | 2576 | A | G | 95 | missense | MeVgp2 | rna-MeVgp2 | c.770A>G | p.Lys257Arg | V protein |
| NC_001498.1 | 2630 | G | T | 122 | missense | MeVgp2 | rna-MeVgp2 | c.824G>T | p.Cys275Phe | V protein |
| NC_001498.1 | 1879 | G | A | 57 | synonymous | MeVgp2 | rna-MeVgp2 | c.51G>A | p.Ser17Ser | C protein |
| NC_001498.1 | 1895 | A | G | 133 | missense | MeVgp2 | rna-MeVgp2 | c.67A>G | p.Arg23Gly | C protein |
| NC_001498.1 | 1938 | A | G | 219 | missense | MeVgp2 | rna-MeVgp2 | c.110A>G | p.Asp37Gly | C protein |
| NC_001498.1 | 1944 | C | G | 213 | missense | MeVgp2 | rna-MeVgp2 | c.116C>G | p.Thr39Ser | C protein |
| NC_001498.1 | 1988 | G | A | 138 | missense | MeVgp2 | rna-MeVgp2 | c.160G>A | p.Ala54Thr | C protein |
| NC_001498.1 | 1993 | C | T | 49 | synonymous | MeVgp2 | rna-MeVgp2 | c.165C>T | p.Asn55Asn | C protein |
| NC_001498.1 | 2017 | A | G | 100 | synonymous | MeVgp2 | rna-MeVgp2 | c.189A>G | p.Gln63Gln | C protein |
| NC_001498.1 | 2167 | A | G | 146 | synonymous | MeVgp2 | rna-MeVgp2 | c.339A>G | p.Leu113Leu | C protein |
| NC_001498.1 | 2201 | A | G | 154 | missense | MeVgp2 | rna-MeVgp2 | c.373A>G | p.Asn125Asp | C protein |
| NC_001498.1 | 2256 | C | T | 51 | missense | MeVgp2 | rna-MeVgp2 | c.428C>T | p.Thr143Ile | C protein |
| NC_001498.1 | 1879 | G | A | 57 | missense | MeVgp2 | rna-MeVgp2 | c.73G>A | p.Gly25Ser | phosphoprotein |
| NC_001498.1 | 1895 | A | G | 133 | missense | MeVgp2 | rna-MeVgp2 | c.89A>G | p.Glu30Gly | phosphoprotein |
| NC_001498.1 | 1938 | A | G | 219 | synonymous | MeVgp2 | rna-MeVgp2 | c.132A>G | p.Gly44Gly | phosphoprotein |
| NC_001498.1 | 1944 | C | G | 213 | missense | MeVgp2 | rna-MeVgp2 | c.138C>G | p.Asp46Glu | phosphoprotein |
| NC_001498.1 | 1988 | G | A | 138 | missense | MeVgp2 | rna-MeVgp2 | c.182G>A | p.Ser61Asn | phosphoprotein |
| NC_001498.1 | 1993 | C | T | 49 | missense | MeVgp2 | rna-MeVgp2 | c.187C>T | p.Pro63Ser | phosphoprotein |
| NC_001498.1 | 2017 | A | G | 100 | missense | MeVgp2 | rna-MeVgp2 | c.211A>G | p.Thr71Ala | phosphoprotein |
| NC_001498.1 | 2167 | A | G | 146 | missense | MeVgp2 | rna-MeVgp2 | c.361A>G | p.Lys121Glu | phosphoprotein |
| NC_001498.1 | 2201 | A | G | 154 | missense | MeVgp2 | rna-MeVgp2 | c.395A>G | p.Gln132Arg | phosphoprotein |
| NC_001498.1 | 2256 | C | T | 51 | synonymous | MeVgp2 | rna-MeVgp2 | c.450C>T | p.Asn150Asn | phosphoprotein |
| NC_001498.1 | 2403 | A | G | 87 | synonymous | MeVgp2 | rna-MeVgp2 | c.597A>G | p.Arg199Arg | phosphoprotein |
| NC_001498.1 | 2576 | A | G | 95 | missense | MeVgp2 | rna-MeVgp2 | c.770A>G | p.Lys257Arg | phosphoprotein |
| NC_001498.1 | 2630 | G | T | 122 | missense | MeVgp2 | rna-MeVgp2 | c.824G>T | p.Cys275Phe | phosphoprotein |
| NC_001498.1 | 2821 | T | C | 40 | synonymous | MeVgp2 | rna-MeVgp2 | c.1015T>C | p.Leu339Leu | phosphoprotein |
| NC_001498.1 | 2830 | T | C | 182 | synonymous | MeVgp2 | rna-MeVgp2 | c.1024T>C | p.Leu342Leu | phosphoprotein |
| NC_001498.1 | 2935 | C | T | 73 | missense | MeVgp2 | rna-MeVgp2 | c.1129C>T | p.Leu377Phe | phosphoprotein |
| NC_001498.1 | 2951 | A | G | 11 | missense | MeVgp2 | rna-MeVgp2 | c.1145A>G | p.Asn382Ser | phosphoprotein |
| NC_001498.1 | 3033 | T | C | 47 | synonymous | MeVgp2 | rna-MeVgp2 | c.1227T>C | p.Val409Val | phosphoprotein |
| NC_001498.1 | 3075 | T | A | 50 | synonymous | MeVgp2 | rna-MeVgp2 | c.1269T>A | p.Thr423Thr | phosphoprotein |
| NC_001498.1 | 3228 | G | T | 40 | missense | MeVgp2 | rna-MeVgp2 | c.1422G>T | p.Glu474Asp | phosphoprotein |
| NC_001498.1 | 3344 | T | C | 89 | . | . | . | . | . |  |
| NC_001498.1 | 3569 | T | C | 80 | synonymous | MeVgp3 | rna-MeVgp3 | c.132T>C | p.Asp44Asp | matrix protein |
| NC_001498.1 | 3740 | T | C | 120 | synonymous | MeVgp3 | rna-MeVgp3 | c.303T>C | p.Val101Val | matrix protein |
| NC_001498.1 | 3797 | C | T | 64 | synonymous | MeVgp3 | rna-MeVgp3 | c.360C>T | p.Thr120Thr | matrix protein |
| NC_001498.1 | 3911 | T | C | 106 | synonymous | MeVgp3 | rna-MeVgp3 | c.474T>C | p.Val158Val | matrix protein |
| NC_001498.1 | 3959 | C | T | 39 | synonymous | MeVgp3 | rna-MeVgp3 | c.522C>T | p.Pro174Pro | matrix protein |
| NC_001498.1 | 3964 | G | A | 86 | missense | MeVgp3 | rna-MeVgp3 | c.527G>A | p.Arg176Lys | matrix protein |
| NC_001498.1 | 4007 | G | A | 150 | synonymous | MeVgp3 | rna-MeVgp3 | c.570G>A | p.Leu190Leu | matrix protein |
| NC_001498.1 | 4010 | A | G | 67 | synonymous | MeVgp3 | rna-MeVgp3 | c.573A>G | p.Leu191Leu | matrix protein |
| NC_001498.1 | 4066 | A | T | 41 | missense | MeVgp3 | rna-MeVgp3 | c.629A>T | p.Glu210Val | matrix protein |
| NC_001498.1 | 4070 | A | G | 93 | synonymous | MeVgp3 | rna-MeVgp3 | c.633A>G | p.Gln211Gln | matrix protein |
| NC_001498.1 | 4112 | G | A | 97 | synonymous | MeVgp3 | rna-MeVgp3 | c.675G>A | p.Arg225Arg | matrix protein |
| NC_001498.1 | 4160 | C | T | 63 | synonymous | MeVgp3 | rna-MeVgp3 | c.723C>T | p.Ile241Ile | matrix protein |
| NC_001498.1 | 4241 | G | A | 28 | synonymous | MeVgp3 | rna-MeVgp3 | c.804G>A | p.Lys268Lys | matrix protein |
| NC_001498.1 | 4244 | T | C | 12 | synonymous | MeVgp3 | rna-MeVgp3 | c.807T>C | p.Thr269Thr | matrix protein |
| NC_001498.1 | 4343 | A | C | 32 | synonymous | MeVgp3 | rna-MeVgp3 | c.906A>C | p.Ile302Ile | matrix protein |
| NC_001498.1 | 4379 | T | C | 34 | synonymous | MeVgp3 | rna-MeVgp3 | c.942T>C | p.Pro314Pro | matrix protein |
| NC_001498.1 | 5344 | C | A | 43 | . | . | . | . | . |  |
| NC_001498.1 | 5351 | T | C | 49 | . | . | . | . | . |  |
| NC_001498.1 | 5383 | T | C | 50 | . | . | . | . | . |  |
| NC_001498.1 | 5517 | A | C | 56 | synonymous | MeVgp4 | rna-MeVgp4 | c.60A>C | p.Thr20Thr | fusion protein |
| NC_001498.1 | 5982 | T | C | 101 | synonymous | MeVgp4 | rna-MeVgp4 | c.525T>C | p.Val175Val | fusion protein |
| NC_001498.1 | 6036 | A | G | 38 | synonymous | MeVgp4 | rna-MeVgp4 | c.579A>G | p.Leu193Leu | fusion protein |
| NC_001498.1 | 6084 | C | T | 99 | synonymous | MeVgp4 | rna-MeVgp4 | c.627C>T | p.Tyr209Tyr | fusion protein |
| NC_001498.1 | 6147 | C | T | 161 | synonymous | MeVgp4 | rna-MeVgp4 | c.690C>T | p.Ser230Ser | fusion protein |
| NC_001498.1 | 6189 | G | A | 39 | synonymous | MeVgp4 | rna-MeVgp4 | c.732G>A | p.Lys244Lys | fusion protein |
| NC_001498.1 | 6192 | A | G | 19 | synonymous | MeVgp4 | rna-MeVgp4 | c.735A>G | p.Val245Val | fusion protein |
| NC_001498.1 | 6387 | G | A | 36 | synonymous | MeVgp4 | rna-MeVgp4 | c.930G>A | p.Glu310Glu | fusion protein |
| NC_001498.1 | 6522 | C | T | 108 | synonymous | MeVgp4 | rna-MeVgp4 | c.1065C>T | p.Leu355Leu | fusion protein |
| NC_001498.1 | 6579 | T | C | 30 | synonymous | MeVgp4 | rna-MeVgp4 | c.1122T>C | p.Ser374Ser | fusion protein |
| NC_001498.1 | 6621 | C | T | 51 | synonymous | MeVgp4 | rna-MeVgp4 | c.1164C>T | p.Ala388Ala | fusion protein |
| NC_001498.1 | 6987 | C | T | 68 | synonymous | MeVgp4 | rna-MeVgp4 | c.1530C>T | p.Ile510Ile | fusion protein |
| NC_001498.1 | 6991 | G | A | 48 | missense | MeVgp4 | rna-MeVgp4 | c.1534G>A | p.Ala512Thr | fusion protein |
| NC_001498.1 | 7396 | A | G | 31 | synonymous | MeVgp5 | rna-MeVgp5 | c.126A>G | p.Leu42Leu | hemagglutinin protein |
| NC_001498.1 | 7588 | G | A | 28 | synonymous | MeVgp5 | rna-MeVgp5 | c.318G>A | p.Arg106Arg | hemagglutinin protein |
| NC_001498.1 | 7633 | A | G | 25 | synonymous | MeVgp5 | rna-MeVgp5 | c.363A>G | p.Lys121Lys | hemagglutinin protein |
| NC_001498.1 | 7636 | T | C | 11 | synonymous | MeVgp5 | rna-MeVgp5 | c.366T>C | p.Ile122Ile | hemagglutinin protein |
| NC_001498.1 | 7849 | A | C | 46 | synonymous | MeVgp5 | rna-MeVgp5 | c.579A>C | p.Thr193Thr | hemagglutinin protein |
| NC_001498.1 | 8101 | G | A | 21 | synonymous | MeVgp5 | rna-MeVgp5 | c.831G>A | p.Glu277Glu | hemagglutinin protein |
| NC_001498.1 | 8206 | G | A | 36 | synonymous | MeVgp5 | rna-MeVgp5 | c.936G>A | p.Gly312Gly | hemagglutinin protein |
| NC_001498.1 | 8271 | G | A | 38 | missense | MeVgp5 | rna-MeVgp5 | c.1001G>A | p.Arg334Gln | hemagglutinin protein |
| NC_001498.1 | 8272 | A | T | 33 | synonymous | MeVgp5 | rna-MeVgp5 | c.1002A>T | p.Arg334Arg | hemagglutinin protein |
| NC_001498.1 | 8606 | A | T | 17 | missense | MeVgp5 | rna-MeVgp5 | c.1336A>T | p.Thr446Ser | hemagglutinin protein |
| NC_001498.1 | 8654 | C | T | 65 | synonymous | MeVgp5 | rna-MeVgp5 | c.1384C>T | p.Leu462Leu | hemagglutinin protein |
| NC_001498.1 | 8696 | T | C | 50 | missense | MeVgp5 | rna-MeVgp5 | c.1426T>C | p.Phe476Leu | hemagglutinin protein |
| NC_001498.1 | 8755 | T | C | 124 | synonymous | MeVgp5 | rna-MeVgp5 | c.1485T>C | p.His495His | hemagglutinin protein |
| NC_001498.1 | 8815 | A | G | 81 | synonymous | MeVgp5 | rna-MeVgp5 | c.1545A>G | p.Val515Val | hemagglutinin protein |
| NC_001498.1 | 8955 | T | C | 15 | missense | MeVgp5 | rna-MeVgp5 | c.1685T>C | p.Val562Ala | hemagglutinin protein |
| NC_001498.1 | 8959 | A | C | 37 | synonymous | MeVgp5 | rna-MeVgp5 | c.1689A>C | p.Pro563Pro | hemagglutinin protein |
| NC_001498.1 | 8991 | A | C | 37 | missense | MeVgp5 | rna-MeVgp5 | c.1721A>C | p.Asp574Ala | hemagglutinin protein |
| NC_001498.1 | 8993 | A | C | 19 | missense | MeVgp5 | rna-MeVgp5 | c.1723A>C | p.Lys575Gln | hemagglutinin protein |
| NC_001498.1 | 8997 | A | G | 19 | missense | MeVgp5 | rna-MeVgp5 | c.1727A>G | p.Lys576Arg | hemagglutinin protein |
| NC_001498.1 | 9177 | T | C | 80 | . | . | . | . | . |  |
| NC_001498.1 | 9443 | T | C | 38 | synonymous | MeVgp6 | rna-MeVgp6 | c.210T>C | p.Asn70Asn | large polymerase protein |
| NC_001498.1 | 9455 | C | T | 56 | synonymous | MeVgp6 | rna-MeVgp6 | c.222C>T | p.Ser74Ser | large polymerase protein |
| NC_001498.1 | 9461 | T | C | 76 | synonymous | MeVgp6 | rna-MeVgp6 | c.228T>C | p.Leu76Leu | large polymerase protein |
| NC_001498.1 | 9503 | T | C | 91 | synonymous | MeVgp6 | rna-MeVgp6 | c.270T>C | p.Cys90Cys | large polymerase protein |
| NC_001498.1 | 9542 | A | G | 67 | synonymous | MeVgp6 | rna-MeVgp6 | c.309A>G | p.Thr103Thr | large polymerase protein |
| NC_001498.1 | 9615 | C | T | 120 | synonymous | MeVgp6 | rna-MeVgp6 | c.382C>T | p.Leu128Leu | large polymerase protein |
| NC_001498.1 | 9623 | C | T | 118 | synonymous | MeVgp6 | rna-MeVgp6 | c.390C>T | p.Asp130Asp | large polymerase protein |
| NC_001498.1 | 9731 | G | A | 45 | synonymous | MeVgp6 | rna-MeVgp6 | c.498G>A | p.Leu166Leu | large polymerase protein |
| NC_001498.1 | 9788 | T | G | 76 | synonymous | MeVgp6 | rna-MeVgp6 | c.555T>G | p.Thr185Thr | large polymerase protein |
| NC_001498.1 | 9878 | G | A | 37 | synonymous | MeVgp6 | rna-MeVgp6 | c.645G>A | p.Lys215Lys | large polymerase protein |
| NC_001498.1 | 9905 | G | A | 102 | synonymous | MeVgp6 | rna-MeVgp6 | c.672G>A | p.Thr224Thr | large polymerase protein |
| NC_001498.1 | 9962 | C | A | 80 | synonymous | MeVgp6 | rna-MeVgp6 | c.729C>A | p.Thr243Thr | large polymerase protein |
| NC_001498.1 | 10025 | G | A | 29 | synonymous | MeVgp6 | rna-MeVgp6 | c.792G>A | p.Leu264Leu | large polymerase protein |
| NC_001498.1 | 10067 | A | G | 84 | synonymous | MeVgp6 | rna-MeVgp6 | c.834A>G | p.Gln278Gln | large polymerase protein |
| NC_001498.1 | 10106 | G | A | 42 | synonymous | MeVgp6 | rna-MeVgp6 | c.873G>A | p.Leu291Leu | large polymerase protein |
| NC_001498.1 | 10109 | A | G | 68 | synonymous | MeVgp6 | rna-MeVgp6 | c.876A>G | p.Gln292Gln | large polymerase protein |
| NC_001498.1 | 10176 | G | A | 11 | missense | MeVgp6 | rna-MeVgp6 | c.943G>A | p.Val315Ile | large polymerase protein |
| NC_001498.1 | 10271 | A | G | 39 | synonymous | MeVgp6 | rna-MeVgp6 | c.1038A>G | p.Thr346Thr | large polymerase protein |
| NC_001498.1 | 10364 | T | C | 31 | synonymous | MeVgp6 | rna-MeVgp6 | c.1131T>C | p.Pro377Pro | large polymerase protein |
| NC_001498.1 | 10491 | A | C | 39 | missense | MeVgp6 | rna-MeVgp6 | c.1258A>C | p.Thr420Pro | large polymerase protein |
| NC_001498.1 | 10559 | A | C | 31 | synonymous | MeVgp6 | rna-MeVgp6 | c.1326A>C | p.Ser442Ser | large polymerase protein |
| NC_001498.1 | 10706 | G | A | 23 | synonymous | MeVgp6 | rna-MeVgp6 | c.1473G>A | p.Lys491Lys | large polymerase protein |
| NC_001498.1 | 10784 | C | T | 82 | synonymous | MeVgp6 | rna-MeVgp6 | c.1551C>T | p.Val517Val | large polymerase protein |
| NC_001498.1 | 10787 | A | G | 34 | synonymous | MeVgp6 | rna-MeVgp6 | c.1554A>G | p.Val518Val | large polymerase protein |
| NC_001498.1 | 10796 | C | T | 26 | synonymous | MeVgp6 | rna-MeVgp6 | c.1563C>T | p.Ala521Ala | large polymerase protein |
| NC_001498.1 | 10868 | T | G | 42 | synonymous | MeVgp6 | rna-MeVgp6 | c.1635T>G | p.Leu545Leu | large polymerase protein |
| NC_001498.1 | 10871 | C | T | 20 | synonymous | MeVgp6 | rna-MeVgp6 | c.1638C>T | p.Phe546Phe | large polymerase protein |
| NC_001498.1 | 10943 | G | A | 27 | synonymous | MeVgp6 | rna-MeVgp6 | c.1710G>A | p.Lys570Lys | large polymerase protein |
| NC_001498.1 | 11006 | G | A | 53 | synonymous | MeVgp6 | rna-MeVgp6 | c.1773G>A | p.Leu591Leu | large polymerase protein |
| NC_001498.1 | 11039 | A | G | 50 | synonymous | MeVgp6 | rna-MeVgp6 | c.1806A>G | p.Glu602Glu | large polymerase protein |
| NC_001498.1 | 11051 | G | A | 39 | synonymous | MeVgp6 | rna-MeVgp6 | c.1818G>A | p.Gly606Gly | large polymerase protein |
| NC_001498.1 | 11204 | C | T | 23 | synonymous | MeVgp6 | rna-MeVgp6 | c.1971C>T | p.Ser657Ser | large polymerase protein |
| NC_001498.1 | 11252 | T | C | 33 | synonymous | MeVgp6 | rna-MeVgp6 | c.2019T>C | p.Tyr673Tyr | large polymerase protein |
| NC_001498.1 | 11255 | G | A | 13 | synonymous | MeVgp6 | rna-MeVgp6 | c.2022G>A | p.Glu674Glu | large polymerase protein |
| NC_001498.1 | 11348 | C | G | 181 | synonymous | MeVgp6 | rna-MeVgp6 | c.2115C>G | p.Leu705Leu | large polymerase protein |
| NC_001498.1 | 11396 | G | A | 183 | synonymous | MeVgp6 | rna-MeVgp6 | c.2163G>A | p.Pro721Pro | large polymerase protein |
| NC_001498.1 | 11450 | A | C | 235 | synonymous | MeVgp6 | rna-MeVgp6 | c.2217A>C | p.Ile739Ile | large polymerase protein |
| NC_001498.1 | 11495 | C | T | 314 | synonymous | MeVgp6 | rna-MeVgp6 | c.2262C>T | p.Tyr754Tyr | large polymerase protein |
| NC_001498.1 | 11507 | T | A | 111 | synonymous | MeVgp6 | rna-MeVgp6 | c.2274T>A | p.Ala758Ala | large polymerase protein |
| NC_001498.1 | 11582 | A | G | 48 | synonymous | MeVgp6 | rna-MeVgp6 | c.2349A>G | p.Val783Val | large polymerase protein |
| NC_001498.1 | 11624 | T | C | 65 | synonymous | MeVgp6 | rna-MeVgp6 | c.2391T>C | p.Ala797Ala | large polymerase protein |
| NC_001498.1 | 11750 | T | C | 36 | synonymous | MeVgp6 | rna-MeVgp6 | c.2517T>C | p.Asp839Asp | large polymerase protein |
| NC_001498.1 | 11798 | C | T | 83 | synonymous | MeVgp6 | rna-MeVgp6 | c.2565C>T | p.Phe855Phe | large polymerase protein |
| NC_001498.1 | 11891 | T | C | 64 | synonymous | MeVgp6 | rna-MeVgp6 | c.2658T>C | p.Tyr886Tyr | large polymerase protein |
| NC_001498.1 | 11930 | A | G | 42 | synonymous | MeVgp6 | rna-MeVgp6 | c.2697A>G | p.Gln899Gln | large polymerase protein |
| NC_001498.1 | 11934 | T | C | 97 | synonymous | MeVgp6 | rna-MeVgp6 | c.2701T>C | p.Leu901Leu | large polymerase protein |
| NC_001498.1 | 11975 | A | G | 106 | synonymous | MeVgp6 | rna-MeVgp6 | c.2742A>G | p.Arg914Arg | large polymerase protein |
| NC_001498.1 | 12008 | T | C | 94 | synonymous | MeVgp6 | rna-MeVgp6 | c.2775T>C | p.Asp925Asp | large polymerase protein |
| NC_001498.1 | 12062 | G | T | 94 | synonymous | MeVgp6 | rna-MeVgp6 | c.2829G>T | p.Leu943Leu | large polymerase protein |
| NC_001498.1 | 12139 | T | A | 71 | missense | MeVgp6 | rna-MeVgp6 | c.2906T>A | p.Leu969His | large polymerase protein |
| NC_001498.1 | 12140 | C | T | 63 | synonymous | MeVgp6 | rna-MeVgp6 | c.2907C>T | p.Leu969Leu | large polymerase protein |
| NC_001498.1 | 12248 | C | T | 86 | synonymous | MeVgp6 | rna-MeVgp6 | c.3015C>T | p.Cys1005Cys | large polymerase protein |
| NC_001498.1 | 12297 | C | T | 87 | synonymous | MeVgp6 | rna-MeVgp6 | c.3064C>T | p.Leu1022Leu | large polymerase protein |
| NC_001498.1 | 12299 | A | G | 41 | synonymous | MeVgp6 | rna-MeVgp6 | c.3066A>G | p.Leu1022Leu | large polymerase protein |
| NC_001498.1 | 12371 | G | T | 56 | synonymous | MeVgp6 | rna-MeVgp6 | c.3138G>T | p.Ala1046Ala | large polymerase protein |
| NC_001498.1 | 12377 | C | T | 102 | synonymous | MeVgp6 | rna-MeVgp6 | c.3144C>T | p.Phe1048Phe | large polymerase protein |
| NC_001498.1 | 12425 | G | A | 57 | synonymous | MeVgp6 | rna-MeVgp6 | c.3192G>A | p.Leu1064Leu | large polymerase protein |
| NC_001498.1 | 12431 | T | C | 16 | synonymous | MeVgp6 | rna-MeVgp6 | c.3198T>C | p.His1066His | large polymerase protein |
| NC_001498.1 | 12515 | G | A | 10 | synonymous | MeVgp6 | rna-MeVgp6 | c.3282G>A | p.Gly1094Gly | large polymerase protein |
| NC_001498.1 | 12539 | A | G | 107 | synonymous | MeVgp6 | rna-MeVgp6 | c.3306A>G | p.Arg1102Arg | large polymerase protein |
| NC_001498.1 | 12617 | C | T | 338 | synonymous | MeVgp6 | rna-MeVgp6 | c.3384C>T | p.Asp1128Asp | large polymerase protein |
| NC_001498.1 | 12629 | T | C | 85 | synonymous | MeVgp6 | rna-MeVgp6 | c.3396T>C | p.Cys1132Cys | large polymerase protein |
| NC_001498.1 | 12806 | T | C | 61 | synonymous | MeVgp6 | rna-MeVgp6 | c.3573T>C | p.Phe1191Phe | large polymerase protein |
| NC_001498.1 | 12953 | A | G | 113 | synonymous | MeVgp6 | rna-MeVgp6 | c.3720A>G | p.Arg1240Arg | large polymerase protein |
| NC_001498.1 | 12992 | T | C | 101 | synonymous | MeVgp6 | rna-MeVgp6 | c.3759T>C | p.Asp1253Asp | large polymerase protein |
| NC_001498.1 | 13100 | G | A | 38 | synonymous | MeVgp6 | rna-MeVgp6 | c.3867G>A | p.Arg1289Arg | large polymerase protein |
| NC_001498.1 | 13175 | C | T | 31 | synonymous | MeVgp6 | rna-MeVgp6 | c.3942C>T | p.Ser1314Ser | large polymerase protein |
| NC_001498.1 | 13178 | C | T | 16 | synonymous | MeVgp6 | rna-MeVgp6 | c.3945C>T | p.Asn1315Asn | large polymerase protein |
| NC_001498.1 | 13196 | C | T | 20 | synonymous | MeVgp6 | rna-MeVgp6 | c.3963C>T | p.Val1321Val | large polymerase protein |
| NC_001498.1 | 13232 | C | T | 40 | synonymous | MeVgp6 | rna-MeVgp6 | c.3999C>T | p.Tyr1333Tyr | large polymerase protein |
| NC_001498.1 | 13272 | T | C | 12 | synonymous | MeVgp6 | rna-MeVgp6 | c.4039T>C | p.Leu1347Leu | large polymerase protein |
| NC_001498.1 | 13307 | C | T | 45 | synonymous | MeVgp6 | rna-MeVgp6 | c.4074C>T | p.Asn1358Asn | large polymerase protein |
| NC_001498.1 | 13382 | C | T | 119 | synonymous | MeVgp6 | rna-MeVgp6 | c.4149C>T | p.Ser1383Ser | large polymerase protein |
| NC_001498.1 | 13391 | A | G | 95 | synonymous | MeVgp6 | rna-MeVgp6 | c.4158A>G | p.Leu1386Leu | large polymerase protein |
| NC_001498.1 | 13439 | A | C | 55 | synonymous | MeVgp6 | rna-MeVgp6 | c.4206A>C | p.Ala1402Ala | large polymerase protein |
| NC_001498.1 | 13502 | A | G | 143 | synonymous | MeVgp6 | rna-MeVgp6 | c.4269A>G | p.Glu1423Glu | large polymerase protein |
| NC_001498.1 | 13574 | G | A | 89 | synonymous | MeVgp6 | rna-MeVgp6 | c.4341G>A | p.Leu1447Leu | large polymerase protein |
| NC_001498.1 | 13625 | G | A | 34 | synonymous | MeVgp6 | rna-MeVgp6 | c.4392G>A | p.Gly1464Gly | large polymerase protein |
| NC_001498.1 | 13830 | C | T | 15 | synonymous | MeVgp6 | rna-MeVgp6 | c.4597C>T | p.Leu1533Leu | large polymerase protein |
| NC_001498.1 | 13832 | A | G | 41 | synonymous | MeVgp6 | rna-MeVgp6 | c.4599A>G | p.Leu1533Leu | large polymerase protein |
| NC_001498.1 | 13874 | T | C | 49 | synonymous | MeVgp6 | rna-MeVgp6 | c.4641T>C | p.Ile1547Ile | large polymerase protein |
| NC_001498.1 | 13889 | T | C | 100 | synonymous | MeVgp6 | rna-MeVgp6 | c.4656T>C | p.His1552His | large polymerase protein |
| NC_001498.1 | 14076 | C | T | 23 | synonymous | MeVgp6 | rna-MeVgp6 | c.4843C>T | p.Leu1615Leu | large polymerase protein |
| NC_001498.1 | 14181 | T | C | 103 | synonymous | MeVgp6 | rna-MeVgp6 | c.4948T>C | p.Leu1650Leu | large polymerase protein |
| NC_001498.1 | 14258 | T | A | 33 | synonymous | MeVgp6 | rna-MeVgp6 | c.5025T>A | p.Arg1675Arg | large polymerase protein |
| NC_001498.1 | 14264 | A | G | 18 | synonymous | MeVgp6 | rna-MeVgp6 | c.5031A>G | p.Gly1677Gly | large polymerase protein |
| NC_001498.1 | 14267 | T | G | 12 | synonymous | MeVgp6 | rna-MeVgp6 | c.5034T>G | p.Ser1678Ser | large polymerase protein |
| NC_001498.1 | 14510 | A | G | 15 | synonymous | MeVgp6 | rna-MeVgp6 | c.5277A>G | p.Leu1759Leu | large polymerase protein |
| NC_001498.1 | 14585 | C | T | 23 | synonymous | MeVgp6 | rna-MeVgp6 | c.5352C>T | p.Gly1784Gly | large polymerase protein |
| NC_001498.1 | 14714 | T | C | 27 | synonymous | MeVgp6 | rna-MeVgp6 | c.5481T>C | p.Tyr1827Tyr | large polymerase protein |
| NC_001498.1 | 14774 | C | A | 41 | synonymous | MeVgp6 | rna-MeVgp6 | c.5541C>A | p.Leu1847Leu | large polymerase protein |
| NC_001498.1 | 14811 | A | G | 70 | missense | MeVgp6 | rna-MeVgp6 | c.5578A>G | p.Ile1860Val | large polymerase protein |
| NC_001498.1 | 14870 | T | C | 68 | synonymous | MeVgp6 | rna-MeVgp6 | c.5637T>C | p.His1879His | large polymerase protein |
| NC_001498.1 | 14960 | A | G | 108 | synonymous | MeVgp6 | rna-MeVgp6 | c.5727A>G | p.Lys1909Lys | large polymerase protein |
| NC_001498.1 | 15005 | T | C | 106 | synonymous | MeVgp6 | rna-MeVgp6 | c.5772T>C | p.Asp1924Asp | large polymerase protein |
| NC_001498.1 | 15146 | A | G | 45 | synonymous | MeVgp6 | rna-MeVgp6 | c.5913A>G | p.Glu1971Glu | large polymerase protein |
| NC_001498.1 | 15152 | C | T | 68 | synonymous | MeVgp6 | rna-MeVgp6 | c.5919C>T | p.Ile1973Ile | large polymerase protein |
| NC_001498.1 | 15203 | T | C | 56 | synonymous | MeVgp6 | rna-MeVgp6 | c.5970T>C | p.Gly1990Gly | large polymerase protein |
| NC_001498.1 | 15386 | A | G | 11 | synonymous | MeVgp6 | rna-MeVgp6 | c.6153A>G | p.Ser2051Ser | large polymerase protein |
| NC_001498.1 | 15452 | C | T | 40 | synonymous | MeVgp6 | rna-MeVgp6 | c.6219C>T | p.Asn2073Asn | large polymerase protein |
| NC_001498.1 | 15659 | A | G | 57 | synonymous | MeVgp6 | rna-MeVgp6 | c.6426A>G | p.Leu2142Leu | large polymerase protein |
| NC_001498.1 | 15707 | G | A | 282 | synonymous | MeVgp6 | rna-MeVgp6 | c.6474G>A | p.Glu2158Glu | large polymerase protein |
| NC_001498.1 | 15749 | C | T | 178 | synonymous | MeVgp6 | rna-MeVgp6 | c.6516C>T | p.Tyr2172Tyr | large polymerase protein |
| NC_001498.1 | 15829 | C | T | 23 | . | . | . | . | . |  |

**Reference**

1. Wang H, Zhang Y, Mao N, Zhu Z, Cui A, Xu S*, et al.* Molecular characterization of measles viruses in China: Circulation dynamics of the endemic H1 genotype from 2011 to 2017. PLoS One **2019**;14:e0218782
